# Impact of Pre-Processed Chickpea Flour Incorporation into “*Mankoushe*” on Appetite Hormones and Scores

**DOI:** 10.3390/foods7100173

**Published:** 2018-10-19

**Authors:** Sahar Dandachy, Hiba Mawlawi, Marwan Chedid, Carla El-Mallah, Omar Obeid

**Affiliations:** 1 Department of Nutrition, Faculty of Public Health, Lebanese University, Tripoli, Lebanon; sahardandashi@hotmail.com (S.D.); himawlawi@yahoo.com (H.M.); 2 Doctoral School of Science & Technology, Lebanese University, Tripoli, Lebanon; 3 Laboratory Department, New Mazloum Hospital, Tripoli, Lebanon; drchedid@gmail.com; 4 Department of Nutrition and Food Sciences, Faculty of Agricultural and Food Sciences, American University of Beirut, P.O. Box 11-0236, Beirut, Lebanon; cae14@mail.aub.edu

**Keywords:** chickpea flour, glycemia, lipidemia, insulin, GLP-1, ghrelin

## Abstract

Recently, there has been an increasing interest in integrating pulse flours into pastries and baked products to improve their nutritional and health benefits. “*Mankoushe*,” a popular Lebanese pastry made up of refined wheat flour was enriched with chickpea flour that is of better nutritional value, and its postprandial glycemia, insulinemia, lipidemia and appetite measures were monitored. A randomized cross-over study was performed on sixteen healthy Lebanese females, age (years): 22.90 ± 3.00, and BMI (kg/m^2^): 22.70 ± 2.65. Over-night fasted females were asked to consume two iso-energetic meals (201 g; 681 kcal) on two separate days, three days apart. One meal was the “*Regular Mankoushe*” (RM) made with white flour 100%, and the second meal was the “*Chickpeas Mankoushe*” (CM) made with a mixture of wheat/chickpea flour (70/30). Blood samples were collected 15 min before meal ingest and at 30, 90, 150 and 210 min postprandial. Glucose, insulin, triglycerides (TG), ghrelin, and glucagon-like peptide 1 (GLP-1) plasma levels were measured. Subjective appetite rating and food intake were also assessed. Incorporation of pre-processed chickpea flour into “*Mankoushe*” as 30% of the dough was associated with a modest reduction in both glucose and insulin levels, and TG was minimally affected. At the level of appetite hormones, changes in GLP-1 were similar, whereas the reduction in ghrelin was significantly lower after the RM meal and thus favored a higher satiating effect compared to CM. This was not paralleled by a similar change in subjective appetite scores and subsequent energy intake. In conclusion, findings suggest that pre-processed chickpea flour could be a promising functional ingredient of traditional pastries to improve their nutritional quality. Nevertheless, further investigations are warranted regarding its satiating effect.

## 1. Introduction

Obesity, nowadays, is recognized as a worldwide epidemic being prevalent among adults as well as among adolescents and children [1,2]. Globally, obesity has shown a striking rise over the past four decades. Referring to the World Health Organization (WHO) estimate data 2016, around 13.1% of the world’s adult population aged 18 years and above were obese (11.1% of men and 15.1% of women). Regarding adolescents and children, 18% of the population aged between 5 and 19 years were overweight or obese in 2016. The WHO estimations for the same year showed that 31% of the Lebanese population aged 18 years and above were obese (27% of men and 35% of women). In Lebanon, obesity appeared to be prevalent in females more than males [3]. Obesity is the most common nutrition-related disorder and a primary universal health concern associated with a high risk of metabolic and chronic diseases, mainly diabetes mellitus, hypertension, cardiovascular and cerebrovascular diseases, reproductive diseases and even some types of cancer [4]. This global epidemic results from an interaction between genetic, environmental and psychosocial factors; however, it is well-known to be mediated by an imbalance between the energy intake and the energy expenditure. For years, there was an increasing interest in foods that regulate appetite, hence food intake, and improve metabolic regulation.

Pulses were a subject of concern, being among foods associated with a reduction in the risk of obesity [5]. Pulses are the non-oily edible seeds of legumes that are harvested only for their dry grains [6]. These grain legumes represent good sources of nutrients and bioactive compounds [7] that modulate the physiological responses which facilitate obesity [5,7]. Pulses are considered low glycemic index foods [8] due to their high dietary fiber content [6]. Their intake has been related to a reduced risk of chronic diseases [7,9]. Therefore, growing efforts are being conducted to integrate pulse flours into composite flours. Composite flours are mixtures of flours made up by replacing entirely or partially wheat flour with flours from other cereals, pulses or tubers (root vegetables). These blends are mainly used either to enhance the nutritive value of food products or to encourage the use of locally grown crops.

Chickpea (*Cicer arietinum* L.) as a pulse flour receives attention nowadays because of its nutritional value [10]. It is a plentiful source of proteins and total amino acids, notably lysine. This property makes chickpea flour an excellent enhancer of protein quality when mixed with other cereal flours known to be low in lysine but rich in sulfur amino acids [11]. Chickpea flour is considered to be a good source of fibers and fat, primarily monounsaturated fatty acids (MUFA), and polyunsaturated fatty acids (PUFA), compared to wheat flour. It is reported to be an important source of minerals and vitamins, mainly vitamin B-complex. Also, it includes some bioactive phenolic compounds [12]. Flour from chickpea was among the most predominant studied pulse flours in different types of food: bread [13], spaghetti [14], cakes [15], biscuits and cookies [16,17]. The growing body of research on the health benefits of chickpea flour has increased. Clinical trials investigating the hypoglycemic properties of chickpea flour generated valuable data. A reduction in the glycemic response was observed when adding raw chickpea flour to whole-wheat flour in bread [13]. However, the reduction was modest when extruded chickpea flour was added to white flour in bread [18]. The effect of chickpea flour on postprandial insulin and subjective appetite has not been confirmed yet. Earlier studies used either chickpea flour made from raw ground grains [13] or extruded flour [18]. Nevertheless, chickpea flour made by other cooking methods was not previously used in such clinical trials.

Chickpeas are available grains in Lebanon, used in several traditional dishes as whole or mashed boiled grains but not as a flour. Boiling is known to enhance the flavor of pulses [19] and increase the availability of healthy nutrients [20] and fiber content [21]. Hence, chickpea flour made by means of boiling grains may be a valuable functional ingredient of traditional Lebanese pastries and baked products. At present, the metabolic properties of pre-processed chickpea flour and its impact on appetite-related hormones have not been previously evaluated.

Thus, this study was designed to incorporate pre-processed chickpea flour made by soaking, boiling and drying grains into a popular Lebanese pastry “*Mankoushe Zaatar*”, to investigate the impact of this incorporation on postprandial glycemia, insulinemia, and lipidemia, and to determine its capacity in modulating the gastrointestinal hormones involved in appetite control, glucagon-like peptide 1 (GLP-1) and ghrelin, in healthy Lebanese females.

## 2. Materials and Methods

### 2.1. Subjects

Healthy female (*n* = 16) subjects aged between 20 and 40 years, having BMI between 18.5 and 29.9 kg/m^2^, were recruited through personal contacts and advertisements at the Doctoral School of Sciences and Technology (DSST), Lebanese University. There were no dropouts during the study period.

### 2.2. Test Meals

“*Mankoushe*”, is a widely consumed Lebanese pastry similar to pizza, in which a refined wheat flour dough-base is topped with mixed herbs, known as “*Zaatar*”.

Refined wheat flour (Crown Flour Mills, Beirut, Lebanon) and pre-processed chickpea flour were used. Chickpea grains were soaked in distilled water in the ratio of 1:10 (*w*/*v*) for 17 h; water was discarded and fresh tap water was added to the chickpeas in the ratio of 1:10 (*w*/*v*). Grains were boiled at 100 °C for 1 h, drained, then dried in an industrial air drier (Tecmon s.r.l; Cassina De’ Pecchi MI, Italy) for 8 h at a temperature of 38–40 °C. Dried grains were ground into powder form with an electrical multifunction swing type portable grinder (TIMEMORE, Shanghai, China), and sifted through a 60-mesh stainless-steel sieve.

Two types of meal were prepared: “*Regular Mankoushe*” (RM) having the dough made up with 100% refined wheat flour and “*Chickpea Mankoushe*” (CM) having the dough made up with a mixture refined wheat/pre-processed chickpea flour (70/30). The wheat replacement rate was based on the amount of pre-processed chickpea flour that could be added and still produce a CM with similar organoleptic properties to RM. Based on a sensory test previous to the study (unpublished data, 2017), a higher incorporation rate of chickpea flour appeared to alter the organoleptic qualities of the end-product.

Doughs were prepared under controlled conditions, and 160 g dough was used for the two test meals. The topping “*Zaatar*” was a mixture of ground oregano, sesame seeds, sumac spices and salt in the following percentages: 35.86%, 43.04%, 17.93%, and 3.15%. Afterward, sunflower oil was added to “*Zaatar*”. The topping mixture was based on the following percentages: 31.70% “*Zaatar*” and 68.30% oil. Doughs were flattened, portioned, topped with the herbs and oil mixture, and baked for 6 min at 300 °C. Mean caloric content of meals is 680.99 ± 2.46 kcal/meal. Calorie contents of meals were determined based on the United States Department of Agriculture (USDA) database. The composition of test meals consumed at the 681 kcal level is presented in Table 1. The two breakfasts were well-tolerated by the participants, with no complaints regarding the palatability or size of meals.

### 2.3. Experimental Protocol

The study protocol was approved by the Ethics Committee of the DSST, Lebanese University. Anthropometric measurements (weight, height) of fasted consented subjects were taken, and body mass index (BMI), lean body mass (LBM) and percentage body fat (% BF) were determined using the bio-impedance body composition analyzer (GAIA 359 PLUS). The study used a randomized blind cross-over design in which each subject served as her own control. Subjects were tested in the follicular phase and were randomly given two iso-energetic breakfasts (*Regular Mankoushe* (RM), *Chickpeas Mankoushe* (CM)) on two separate days, three days apart. Overnight fasted subjects attended the research center in the morning, were comfortably seated, and a catheter was inserted in the antecubital vein for blood draws. Baseline blood was drawn 15 min before breakfast consumption. Test meals were served allowing 10 min for subjects to finish their breakfast. During the experiment, food and beverages were prohibited, and subjects were not allowed to talk about food, appetite, satiety, and fullness. They were allowed only to use the toilet or to have light entertainments. At 230 min following breakfast, subjects were asked to consume a standardized ad libitum lunch: a pizza meal (525 g; 999.46 kcal). Calorie content of the pizza was determined based on the USDA database [22]. They were instructed to eat at their desire.

### 2.4. Collection and Preparation of Blood Samples

**Figure 1:**
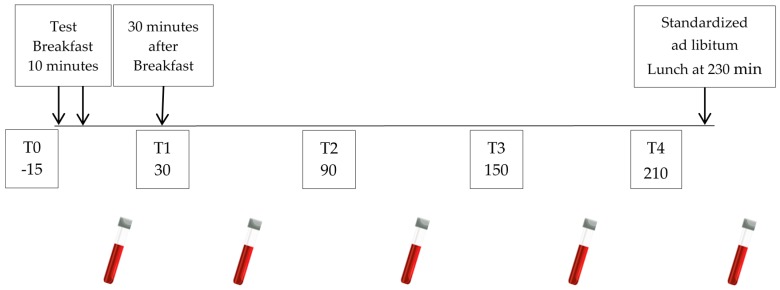
Study design for blood collection and food intake.

Blood samples were collected 15 min before meals (time 0) and postprandial after 30, 90,150, and 210 min (Figure 1) and saline was infused following blood draws to keep the catheter patent. Pefabloc was added at a final concentration of 1 mg/mL in one of the ethylenediamine tetraacetic acid (EDTA) tubes for the stabilization and measurement of plasma ghrelin, since it is a highly unstable hormone. All samples were directly centrifuged at 2500× *g* for 15 min at 5 °C. Plasma samples intended for ghrelin measurements were acidified with HCl to a final concentration of 0.05 N. All specimens were stored at −80 °C until further analysis. None of the 16 subjects experienced any particular discomfort during blood withdrawal.

### 2.5. Biochemical Analysis

Serum glucose and triglycerides (TG) were determined via commercial enzymatic colorimetric tests on the Vitros analyzer (Ektachem DT60 II System, Johnson & Johnson Clinical Diagnostics, Inc., New York, NY, USA). Plasma insulin was measured using a commercially available radioimmunoassay kit (EMD Millipore, Burlington, MA, USA). The lower detection limit was 1 μU/mL when using a 20 μL sample size. Plasma glucagon-like peptide 1 (GLP-1) was measured using a commercially available radioimmunoassay kit (EMD Millipore, Burlington, MA, USA). The lower detection limit was 1.5 pM when using a 50 μL sample size. Plasma total ghrelin was measured using a commercially available radioimmunoassay kit (EMD Millipore, Burlington, MA, USA). The lower detection limit was 50 pg/mL when using a 20 μL sample size.

### 2.6. Subjective Appetite Rating and Food Intake

Subjective appetite information was collected directly after breakfast (time 0) and postprandial at 30, 60, 90,120, 150, 180, and 210 min using the visual analog scale (VAS) questionnaire. A previously validated motivation-to-eat VAS was used [23], and this covered four perceptions: Hunger, Fullness, Satiety and Prospective Food Consumption [24]. Subjects were asked to mark on the line indicating their perception at the designated time points. The magnitude of the subjective feelings was determined by measuring the distance (mm) from the left end starting point to the intersection of the mark on the line [13]. A composite satiety score (CSS) was calculated using the following equation:

CSS = (satiety + fullness + (l00 − prospective food consumption) + (100 − hunger))/4(1)

High CSS is associated with higher satiety perceptions and subsequent lower motivation to eat [25]. Subsequent food intake (FI) was expressed as the amount of consumed pizza.

### 2.7. Statistical Analysis

Data were analyzed using SPSS (Statistical Package for Social Sciences, IBM Statistics version 23). Data were expressed as means ± standard error of mean (SEM), and a *p*-value < 0.05 was considered statistically significant. Data were based on the changes in values from baseline (incremental values). Descriptive summary statistics for mean and SEM were performed for all blood parameters at each time-point for each test meal. Paired *t*-tests were performed to detect changes in variables over time after meal consumption, and to identify differences in the variables’ responses to the two meals at every time point. Results were compared by repeated measures analysis of variance (ANOVA) for main effects of time, test meal and time × meal interaction. Incremental areas under the curve (IAUC) over 210 min were calculated for glucose, insulin and TG changes from baseline for the two meals, using the trapezoidal rule and ignoring the area beneath baseline [26].

## 3. Results and Discussion

This randomized study was designed to incorporate pre-processed chickpea flour into a popular Lebanese pastry and to investigate the impact of this incorporation on postprandial glycemia, insulinemia, lipidemia, selected appetite hormones (ghrelin, GLP-1), appetite score and subsequent energy intake. To our knowledge, the current study is the first study assessing the impact of pre-processed chickpea flour on metabolic parameters and appetite-related hormones.

Subjects’ characteristics at baseline are presented in Table 2. Participants were healthy of a normal weight and overweight with a BMI ranging from 19.7 to 29 kg/m^2^ and having a mean age of 22.9 ± 3.00 years. Participants appeared to have a mean fasting blood glucose below 100 mg/dL and mean insulin level below 30 μU/mL, considered to be in the normal ranges [27,28]. Mean TG level (55.46 ± 17.29 mg/dL) was within the optimal range [27]. Participants showed normal appetite hormones levels (GLP-1 and ghrelin).

### 3.1. Postprandial Changes in Glucose, Insulin, and TG

**Figure 2:**
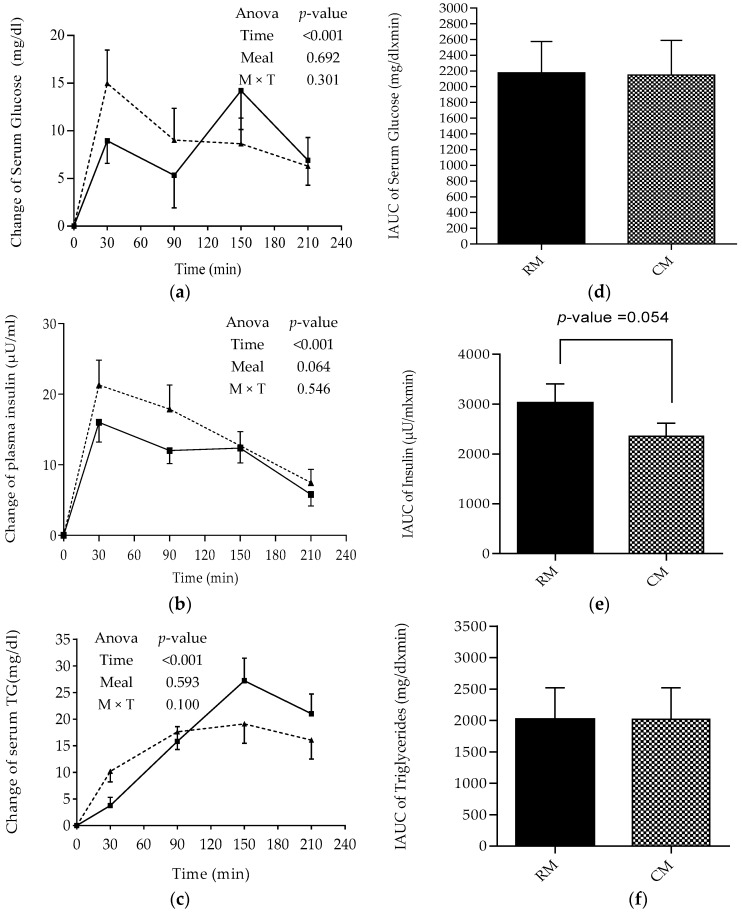
Postprandial changes in glucose (**a**), insulin (**b**) and TG (**c**) from baseline following the ingestion of RM and CM meals. Bars are the incremental areas under the curve (IAUC) of the changes in serum glucose (**d**), insulin (**e**) and triglycerides (TG) (**f**). Data are expressed as mean values ± SEM. RM: *Regular Mankoushe* ---▲---; CM: *Chickpeas Mankoushe* ―■―; M: Meal; T: Time.

Changes in postprandial serum glucose were significantly different according to time (*p* < 0.001), but not a meal and time × meal interaction. However, serum glucose of the CM was slightly lower than that of the RM during the first 90 min, though this failed to reach statistical significance. Moreover, at time 150, serum glucose of the CM showed another peak unlike that of the RM. Nonetheless, IAUC for serum glucose changes calculated over 210 min showed no difference following RM and CM meals (Figure 2a).

Regarding insulin, changes in plasma levels showed a peak at 30 min following both RM and CM meals. The two-way ANOVA with repeated measures presented a significant main effect of time (*p* < 0.001) but not a main meal effect or time × meal interaction. Though plasma insulin level of CM was found to be slightly lower than that of RM during the first 90 min, and in line afterward. Incremental areas under the curve for insulin changes from baseline calculated over 210 min showed near significant (*p* = 0.054) lower values for CM compared to RM (Figure 2b).

In the current study, the peak in glucose level observed 30 min following meals (RM and CM) was associated with a peak in insulin response, and this was reported previously following white and extruded chickpea flour bread [18]. Nevertheless, the peak in glucose level observed 150 min following CM was not seen earlier in any study. The reason behind this peak is unclear. Chickpea flour is considered a low glycemic index food [12] compared to refined wheat flour; however, the peak observed 2 h after CM could be related to intrinsic factors (rate of gastrointestinal motility, digestion, and absorption) and not to food constituents (fibers, fat, and protein) [29].

Findings from the present study showed that incorporation of pre-processed chickpea flour into “*Mankoushe*” as 30% of the dough was associated with a modest reduction, though it failed to reach statistical significance, in both glucose and insulin levels during the first 90 min, as compared with that of RM. Similarly, a trending lower postprandial glycemic response was observed earlier when 25% of the refined wheat flour was replaced by raw or extruded chickpea flour in bread compared to white bread [18]. However, substituting 25 and 35% of whole wheat flour for raw chickpea flour was reported to reduce the glycemic response when compared to white bread [13], and no comparison was made with whole wheat bread. It remains to be understood whether the type of chickpea and its processing, which is known to increase starch digestibility [30] and reduce the content of anti-nutrient substances—tannins, phytic acid, and saponins [31], have impacted the glycemic response. The modest reduction in glucose and insulin level of the CM meal may relate to carbohydrate content (e.g., amylose) of the meal [32] as well as to their high capacity for retrogradation and production of resistant starch [33].

In the current study, the mean changes in TG from baseline were significantly lower at 30 min (*p* = 0.008), comparable at 90 min and higher at 150 and 210 min following CM compared to RM, and the difference was significant at 150 min (*p* = 0.009). Incremental areas under the curve for TG changes from baseline calculated over 210 min showed similar values for both breakfasts. The two-way ANOVA with repeated measures revealed a significant main effect of time (*p* < 0.001) but not a main meal effect or time x meal interaction on serum TG changes from baseline (Figure 2c).

Pulse rich diets have received attention as many researchers suggest that long-term pulse consumption can reduce the risk of cardiovascular diseases (CVD) in humans [7,34]. However, the acute effect of pulse intake on lipid profile has not been extensively studied. Recently, it has been postulated that elevated non-fasting TGs taken randomly are a risk factor for CVD and other chronic diseases [35]. In the current study, postprandial TG was minimally (~30 mg/dL) manipulated, and IAUC were similar between the meals. Findings were in line with other studies, in which no difference in postprandial TG was reported among chickpeas, whole wheat-based meals, and standard meal [36]. Our results show that the TG levels (RM (70.16 mg/dL) and CM (87.09 mg/dL) at time 150 min (peak time)) were below 180 mg/dL, which is considered the desirable non-fasting TG level [35].

### 3.2. Postprandial Changes in Appetite Hormones

**Figure 3:**
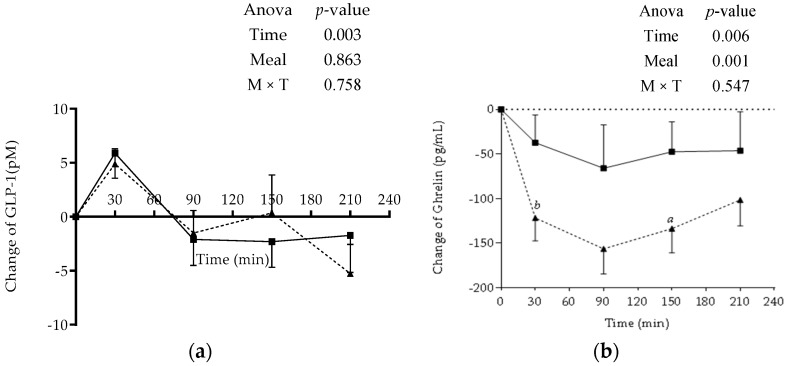
Postprandial changes in plasma glucagon-like peptide 1 (GLP-1) (**a**) and Ghrelin (**b**) from baseline following the ingestion of RM and CM meals. Data are expressed as mean values ± SEM. Values with different letters are significantly different at each time point. ^a^ *p*-value < 0.05, ^b^ *p*-value < 0.01. RM: *Regular Mankoushe* ---▲---; CM: *Chickpeas Mankoushe* ―■―; M: Meal; T: Time.

Glucagon-like peptide 1 changes following the two breakfasts peaked similarly at 30 min and decreased below baseline from time 90 min and onward. The two-way ANOVA with repeated measures revealed a significant main effect of time (*p* = 0.003) but not a main meal effect or time x meal interaction on plasma GLP-1 changes from baseline (Figure 3a).

As for ghrelin, changes in plasma levels show that all values were dropped below baseline; this reached a nadir at 90 min following both breakfasts. The magnitude of reduction was different between the groups. Ghrelin levels were found to be higher at all time points following CM compared to RM, and the difference was significant at 30 min (*p* = 0.006) and 150 min (*p* = 0.043). The two-way ANOVA with repeated measures revealed a significant main effect of time (*p* = 0.006) and meal (*p* = 0.001) but not a main effect of time x meal interaction on the plasma ghrelin changes (Figure 3b).

At the level of appetite hormones, both meals were found to have minimal effect on the anorectic hormone GLP-1 unlike that of the orexigenic gut hormone, ghrelin [37]. To our knowledge, no previous studies were reported earlier regarding the acute impact of chickpea or pulses as flours or as grains on GLP-1 or ghrelin. Other authors studied the impact of different kinds of bread on gastrointestinal hormones, and they have found no differential effect of white breads or wholemeal bread on both peptides (GLP-1 and ghrelin). Our results were in accordance with these previous findings regarding GLP-1 but not ghrelin [24].

The differences seen in our study in ghrelin levels between the meals imply that RM had more satiating effects, though this was not paralleled by similar changes in subjective appetite scores and subsequent energy intake, although complex carbohydrates and proteins are reported to suppress ghrelin secretion [38]. However, this was not observed in the current study with chickpea flour. Nevertheless, changes in ghrelin were inversely associated with changes in insulin, and this is in line with other studies [39].

As for the anorectic hormone GLP-1, it is a gut peptide that shows a biphasic response after meal consumption. The first peak of GLP-1 usually occurs 15 to 30 min postprandial [40]. Moreover, it is is an incretin hormone that plays an additional role in enhancing insulin secretion [41], and this was observed for the first 30 min postprandial in our findings.

### 3.3. Subjective Appetite Rating and Food Intake

**Figure 4:**
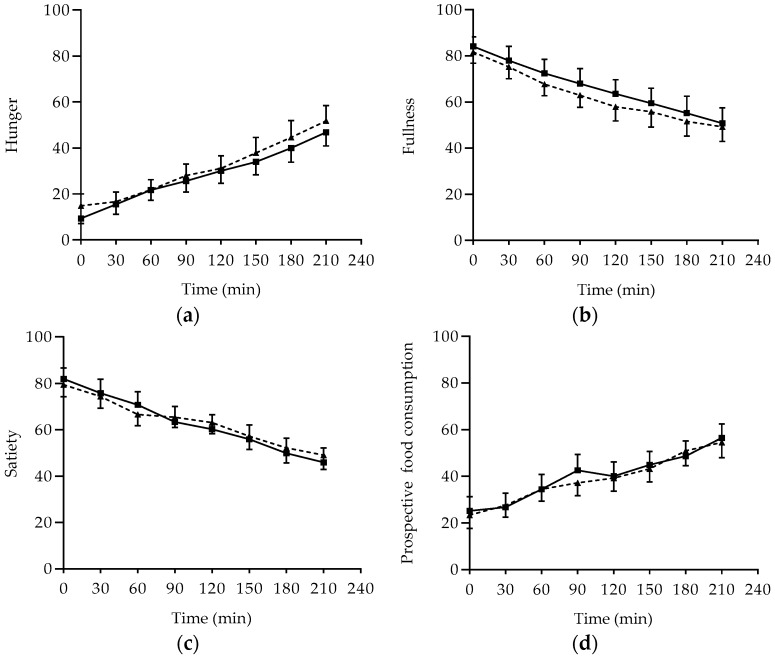
Mean subjective rating for Hunger (**a**), Fullness (**b**), Satiety (**c**), and Prospective food consumption (**d**), following the ingestion of RM and CM meals. Data are expressed as mean values ± SE. RM: *Regular Mankoushe* ---▲---; CM: *Chickpeas Mankoushe* ―■―; M: Meal; T: Time.

**Figure 5:**
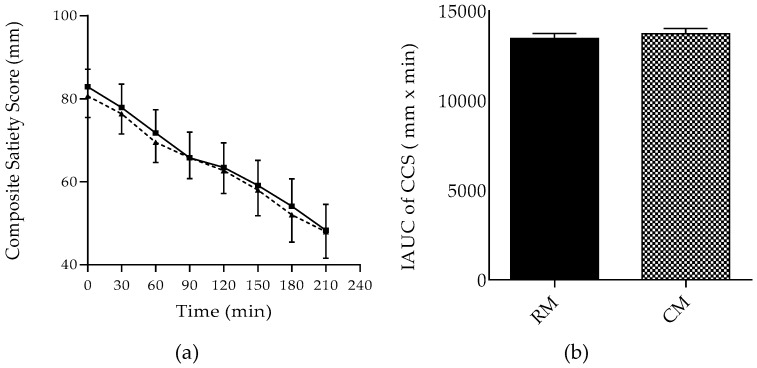
Composite Satiety Score (CSS) changes (**a**) following the ingestion of RM and CM in healthy adult females. *i*AUC of CSS (**b**) following the ingestion of RM and CM meals. Data are expressed as mean values ± SEM. RM: *Regular Mankoushe* ---▲---; CM: *Chickpeas Mankoushe* ―■―; M: Meal; T: Time.

Subjective appetite ratings and CSS values are shown in Figure 4 and Figure 5, respectively. Throughout the experiment, perceptions of hunger and prospective food consumption (PFC) increased, and perceptions of fullness and satiety decreased gradually following the two breakfasts. Within the two breakfasts, the mean values for the four perceptions, Hunger, Fullness, Satiety, and PFC, were comparable (Figure 4). Regarding the CSS, it appeared to decrease similarly following RM and CM, and IAUC for CSS calculated over 210 min showed similar values for both breakfasts (Figure 5). In the present study, almost similar results were noted in the ad libitum pizza meal regarding weight and energy following the two breakfasts. Mean weight and energy content of ad libitum pizza was (233.93 ± 14.94 g, 445.34 ± 20.11 kcal) following RM compared to (232.18 ± 23.78 g, 442 ± 32.02 kcal) following CM. Calorie contents were based on the USDA database [22].

Canned chickpea grains were found to suppress satiety and energy intake from a test meal consumed 120 min after the preload meal [42]. However, chickpea as flour was not found to impact satiety. Our findings of failure to influence measures of appetite scores and subsequent food intake were in line with previous investigations that could not confirm the satiating effect of chickpea flour [13,18]. The difference between chickpea as boiled grains and as flour could be attributed to the particles size. It has been reported that fine flour provides a low satiety response compared to coarse flour [43]. Thus, further investigations regarding the satiating effect of chickpea flour are necessary.

## 4. Conclusions

In conclusion, incorporating pre-processed chickpea flour as 30% into “*Mankoushe”* was found to have a modest reduction in postprandial glycemia and insulinemia. However, it was not found to alter TG level, appetite scores, and subsequent food intake. The treated chickpea flour appeared to differently affect the gut peptides, GLP-1 and ghrelin. Chickpea flour may offer a promising functional ingredient to enhance the nutritional quality of traditional products. Nevertheless, more investigations are warranted regarding the incorporation rate, the processing method, and other appetite-related hormones. Mechanisms mediating gut-peptide regulation are still unclear. Understanding these mechanisms could provide new promising strategies to combat obesity and other metabolic disorders.

## Figures and Tables

**Table 1:** Composition and energy content * of test breakfasts “*Mankoushe*”.

Ingredients	Weight (g)	Regular Mankoushe (kcal)	Chickpea Mankoushe (kcal)
Dough	160	394.38	399.31
Topping	13	44.15	44.15
Oil	28	240	240
Total	201	678.53	683.46

* Energy is based on United States Department of Agriculture (USDA) database [22].

**Table 2:** Baseline characteristics of the study subjects.

Parameter	Mean ± SEM	Range
Age (year)	22.90 ± 3.00	21.0–34.0
Weight (kg)	58.18 ± 6.78	47.1–75.5
BMI (kg/m^2^)	22.70 ± 2.65	19.7–29.0
LBM (kg)	42.90 ± 3.36	36.1–50.5
Body Fat (%)	25.92 ± 3.58	20.1–33.1
Blood Glucose (mg/dL)	88.12 ± 8.16	73–103
Insulin (μU/mL)	4.49 ± 1.68	1.91–9.12
TG (mg/dL)	55.46 ± 17.29	24–100
GLP-1 (pM)	26.29 ± 10.21	13.73–47.08
Ghrelin (pg/mL)	527.28 ± 213.65	253.83–1131.5

Data are expressed as means ± SEM (Standard Error of the Mean); BMI (Body Mass Index); Lean Body Mass (LBM); Triglycerides (TG); Glucagon-like peptide 1 (GLP-1).
